# Health facilities preparedness to deliver maternal and newborn health care in Kilifi and Kisii Counties, Kenya

**DOI:** 10.1186/s12913-023-09884-9

**Published:** 2023-08-16

**Authors:** James Orwa, Marleen Temmerman, Lucy Nyaga, Kennedy Mulama, Stanley Luchters

**Affiliations:** 1 Department of Population Health Sciences, Aga Khan University Kenya, P.O. Box 30270, Nairobi, 00100 Kenya; 2 Department of Public Health and Primary Care, Faculty of Medicine and Health Sciences, Ghent University, Ghent, Belgium; 3 Centre of Excellence for Women and Child Health, Aga Khan University, Nairobi, Kenya; 4 Aga Khan Health Services, Mombasa, Kenya; 5 Aga Khan Health Services, Kisumu, Kenya; 6 Centre for Sexual Health and HIV/AIDS Research (CeSHHAR), Harare, Zimbabwe; 7 Liverpool School of Tropical Medicine (LSTM), Liverpool, UK

**Keywords:** Community health systems, Maternal child health, Quality of care, Preparedness, Obstetric delivery

## Abstract

**Introduction:**

Health facility preparedness is essential for delivering quality maternal and newborn care, minimizing morbidity and mortality by addressing delays in seeking skilled care, reaching appropriate facilities, and receiving emergency care. A rapid assessment of 23 government health facilities in Kilifi and Kisii counties identified poor maternal and newborn indicators in 16 facilities. The Access to Quality Care through Extending and Strengthening Health Systems (AQCESS) project supported these facilities with training, equipment, and referral linkages. This study focuses on facility preparedness of the 16 facilities to deliver maternal and newborn health services, specifically delays two and three at the end of the project implementation.

**Methods:**

A descriptive cross-sectional study was carried-out on behalf of AQCESS project team by respective county ministry of health in-charge of reproductive maternal newborn and child health programs and trained nurses and medical doctors from Aga Khan health services in December 2019. The study evaluated the accessibility and reliability of drugs, commodities, equipment, personnel, basic necessities (such as water and electricity), and guidelines using validated World Health Organization service availability and readiness assessment tool. The findings of the assessment are presented through frequency and percentage analysis, along with a comparative analysis between the two counties.

**Results:**

All the 16 facilities assessed offered routine antenatal care (ANC) and normal delivery, but only two provided comprehensive emergency obstetric and newborn care (CEmONC). Most essential medicines, commodities, and required equipment were available. BEmONC and CEmONC guidelines were present in Kilifi, not in Kisii. One staff member was available 24/7 for cesarean section (CS) in each county, with one anesthetist in Kilifi. Electricity was accessible in all facilities, but only half had secondary power supply. Facilities offering CS had backup generators.

**Conclusion:**

The Facilities assessed had necessary drugs, commodities, equipment, and requirements, but staffing and guidelines were limited. Kilifi outperformed Kisii in most indicators. Additional support is needed for infrastructure and human resources to deliver quality maternal and newborn health services. Continuous monitoring will facilitate resource allocation based on facility needs.

## Introduction

Maternal mortality refers to deaths due to complications from pregnancy or childbirth within 42 days after birth or termination of a pregnancy, not including deaths due to accident or violence [1]. Globally, this has declined from 342 deaths to 211 deaths per 100,000 live births from 2000 to 2017, respectively [2]. Of these deaths, sub-Saharan Africa (SSA) and southern Asia contributed 86% in 2017 of which two-thirds were accounted for by SSA alone. According to the Kenya demographic and health survey report of 2014, maternal mortality ratio (MMR) declined from 520 deaths in 2009 to 362 deaths per 100,000 live births in 2014 [3]. Compared with the neighboring countries in the region, this was lower than Tanzania (MMR of 556 deaths per 100,000 live births in 2016) [4], but higher than neighboring Uganda (MMR of 336 deaths per 100,000 live births in 2016 ([1]). Despite the progress achieved, none of these countries met the Millennium Development Goal 5, and are in the process of making efforts to achieve sustainable development goal 3 of reducing the MMR to 70 deaths per 100,000 live births by 2030 [5].

Postpartum hemorrhage (PPH), hypertension, infections, obstructed labor, and complications from delivery and unsafe abortions are some of the known causes of maternal deaths [6]. Infections, pre-term birth, and intra-partum complications are attributed to the neonatal deaths [7]. Some of these deaths are preventable through effective use of high cost-effective interventions that should be available at the primary care level and also by reducing delays in reaching the facility that provides emergency obstetric care (delay two) and in receiving quality care after arrival at the health facility (delay three) [8]. These delays in addition to delay in seeking care (delay one) have been discussed extensively in the article by Thaddeus and Maine (1994). In brief, the delay in reaching health facility is influenced by transportation availability and affordability, geographical barriers, poor road infrastructure, and lack of emergency transportation services, whilst delay in receiving quality care is influenced by inadequate availability and accessibility of healthcare services, insufficient healthcare workforce, lack of essential medical supplies and equipment, poor quality of care, and financial constraints [9]. The three delays are interlinked with each other and operates in a synergetic manner leading to delay in receiving life-saving interventions, increased complications, and higher maternal and child mortality rates. The study specifically focused on delays two and three.

### Kenya health systems

The Kenya health system is structured in a hierarchical manner that begins with primary healthcare at the lowest level in the community, and then graduates, with complicated cases being referred to higher levels of healthcare. The primary care units consist of community dispensaries, health centers, sub-county teaching and referral facilities, and national hospitals designated as level 1, 2 ,3,4, and 5, respectively under the devolved system of the government [10]. All the levels 1,2,and 3 are expected to provide BEmONC services as the hospital provides CEmONC services according to the WHO recommendations [11, 12]. According to the Kenyan ministry of health sector strategic and investment plan (KHSSP), two nursing staffs are required to serve a population of 10,000 at dispensary levels. At higher level a nurse is required for each ten inpatients, at least two nurses are needed per operating theater table and three nurses are every 24 h of eight-hour shifts each. The distribution of the health care workers within the country is not equitable, resulting in significant health workforce gaps in some of the counties. Details of on how the required healthcare workforce were determined and national health infrastructure norms and standards are provided elsewhere [13, 14].

Under the new Kenyan constitution of 2010, government services including health were devolved with the transfer of power and responsibility for planning, administration and decision-making from national government to county government [15]. The county government determines their own priorities and resource planning, which may compromise national targets to improve maternal and newborn health outcomes. The health service delivery data used in generating health targets at the national level are generated at the local levels, then aggregated at the county levels before transferring to the national level through the national health information system. With the devolution, services of community health volunteers were reinforced to provide basic prevention and health care services including implementing protocols for community based maternal and newborn health at the community level in order to avoid the three delays(delay in deciding to seek care from a skilled attendant by pregnant woman; delay in reaching the facility with capacity to offer basic emergency obstetric care; and delay in receiving emergency care upon reaching a health facility) [16]. In 2013, to further improve maternal and newborn health services, the GoK introduced no charges at all levels of the health facilities for the maternity services as one of the ways to improve delivery services [17] in a country.

Assessment of thirteen counties with the highest maternal and neonatal mortality showed that only 2% of health and dispensaries had essential supplies required for BEmONC signal functions whilst 35% had all the supplies required to provide these functions [16]. The Kenya harmonized health facility assessment found highest scores for availability of equipment and lowest for essential medicines. Among the essential medicines, Gentamicin injection was commonly available and ampicillin powder for injection was least available within the facilities assessed. The report concludes that none of the facilities had all the essential drugs available and only 24% of the health facilities in Kenya had all the basic equipment [18]. Despite the assessment of facilities within counties with the highest maternal and neonatal mortality, a contextual assessment of the level of preparedness in the AQCESS project implementation health facilities is necessary to develop a more nuanced understanding of the health facilities level of preparedness to improve maternal and Newborn health outcomes at the end of the project implementation period.

## Materials and methods

### AQCESS project

AQCESS was a four-year project executed by the Aga Khan Foundation Canada with funding from the Government of Canada. The project aimed to improve reproductive, maternal, newborn and child health (RMNCH) outcomes for women, neonates and children under the age of five and ultimately contribute to the reduction of maternal and child mortality in targeted regions in Kilifi County (Kaloleni and Rabai sub counties) and Kisii County (Bomachoge Borabu Sub County). The project team together with the county ministry of health in-charge of reproductive maternal newborn and child health provided continuous trainings in Emergency Obstetric and Newborn Care (EmONC) signal functions to the health care providers, improved the structures in maternity department of sub-county hospitals, strengthening referral linkages through Community Health Volunteers (CHVs) and provided equipment and supplies in all the selected facilities. Details of other activities implemented by the project are documented elsewhere [19].

### Study design

A descriptive repeat cross-sectional study was conducted in the two rural AQCESS implementation target sub-counties of Kilifi and Kisii counties in August-September 2016 and December 2020. A total of 16 out of the 23 GoK health facilities within the two sub-counties of Kaloleni-Rabai and Bomachoge-Borabu sub-counties of Kilifi and Kisii counties were assessed. The social-cultural characteristics of the study areas are detailed elsewhere in our previous studies in the same settings [20, 21].

### Sample and sampling procedure

Bomachoge-Borabu sub-county in Kisii has a total of 12 health facilities, of which six are owned by the ministry of health, five privately owned and one is faith based. Among the six GoK health facilities, only one is level 4 facility, while the rest are level 2 or 3. In Kaloleni-rabai sub-counties of Kilifi, there are 42 health facilities, of which 17 are owned by GoK and the rest are either private ownership or faith-based.

The sampling procedure used in this survey was non-probability convenience sampling of the 16 targeted health facilities, consisting of six in Kisii and ten in Kilifi counties, where the project was implemented. Following rapid health facility assessments and review of monthly data reports on indicators on maternal, newborn and child health, the team purposively selected the sites in collaboration with the respective county health officials.

### Data collection

The data used in this study were collected as part of an end line survey for AQCESS using a modified questionnaire adapted from service availability and readiness assessment tool by World Health Organization (WHO)[22]. The study analyzed data on antenatal services, delivery services, emergency (BEmONC/CEmoNC) services and infrastructure (water and electricity). Data were provided by the health facility in-charges, the most senior RMNCH personnel, or most knowledgeable health worker about the department or service station who was available at the time of data collection. The data were collected by four trained nurses with a diploma in Kenya registered and community health nursing qualification who assessed the EMoNC signals, observed the validity of the available drugs and functionality of different equipment and supplies available at the facilities. Following the training, two teams consisting of two nurses each were formed (one team in Kisii County and the other in Kilifi). The nurses were not involved in any way with the project implementation. The team was supervised by a qualified medical doctor who ensured the data collection followed the correct procedure. Upon arrival at a facility, the data collection team held brief meetings with facility in-charges and different departmental heads to be assessed giving details of the assessment and the procedure. The survey team used android tablets programmed with the data collection tool in the open data kit platform for data collection. The collected data were reviewed and transmitted to the cloud server at the end of the day’s work. On average, the data collection process lasted for three hours at small facilities (dispensary and some health centers) and six hours for large facilities (sub-county hospitals). All data collection were collected at the department or station where the service was provided.

Ethical clearance to conduct the study was obtained from the Aga Khan University ethics review committee and permits were obtained from NACOSTI approval number:2019/IERC-104(v2). Approval to conduct the study was also obtained from the county and sub-county health officers, and each health facility in-charge gave permission for data collection. All the respondents signed an informed consent before giving any information to the enumerators and were assured of the confidentiality of the information and their right to withdraw at any stage without prejudice.

### Measurements of variables

Variables assessing availability of basic emergency obstetric care (BEmONC)/comprehensive emergency obstetric care (CEmONC) service availability were defined based on the WHO guidelines [16]. According to this guidelines, health facility is classified as CEmONC if they reported performing cesarean sections and blood transfusion, in addition to the seven BEmONC signal functions: parental administration of antibiotics, parental administration of oxytocin, parental administration of anticonvulsants for pre-eclampsia and eclampsia (e.g. Mg_2_SO_4_), perform assisted vaginal delivery (e.g. manual extraction and forceps delivery), perform manual removal of placenta, perform manual removal of retained products of conception (e.g. manual vacuum extraction, dilation and curettage), and perform basic neonatal resuscitation (e.g. with bag and mask) .

The availability of items for obstetric and newborn care were assessed across different domains capturing the aspect of service delivery: (1) equipment, (2) medicines and commodities, (3) general requirements including basic amenities, and (4) staff and guidelines. Specifically, equipment were assessed on the basis of availability and functionality with responses on the availability categorized into observed, reported but not seen and not available, while the functionality at the time of assessment was reported as functioning, not functioning and don’t know. The basic medicine and commodities were assessed using a list of essential medicines for delivery and newborns on their availability and validity. The availability was assessed as observed, reported but not seen, and not available while valid was assessed as valid, invalid, and don’t know. Medication validity was determined by the absence of expiration and adherence to appropriate storage practices.

General facility requirements were assessed in terms of the availability of primary and secondary power supply, primary source of water, availability of a professional who can perform CS 24/7, availability of anesthetics 24/7 in these facilities, availability of functional ambulance, access to ambulance or emergency transport, and availability of fuel for the ambulance. The assessment also included availability of the guidelines on BEmONC and CEmONC. All the assessments were conducted through on-site observations and direct verification.

The theoretical framework of the survey was partly based on the Donabedian model which represents a quality-of-care framework in three interlinked, unidirectional dimensions: structure, process and outcome [23]. In brief, the model proposes that high-quality care is assessed by changes in structure-related items (commodities, equipment and guidelines) which directly influence the process of care (patient diagnosis and treatment) which in turn, has influence on health outcomes (morbidity and mortality) [16].

### Statistical analysis

Descriptive analysis of the different variables was performed using frequency and percentages. The analyses were stratified by the regions and presented as tables showing the frequencies of the health facilities in each county meeting the different criteria in the domains assessed.

## Results

### General characteristics of surveyed facilities

In total, 16 health facilities consisting of 11 dispensaries (level 2), two health centers (level 3) and three sub-county referral hospitals (level 4) were assessed. Kilifi County had six dispensaries, two health centers and two hospitals, whilst Kisii had only one sub-county referral hospital and five dispensaries. All the health facilities were providing normal delivery services and BEmONC services. However, only two were providing CEmONC services. There were three facilities with newborn care units in Kilifi County and none in Kisii (Table 1).

**Table 1:** Delivery services available in health facilities, N = 16

		County	
Services	N = 16	Kilifi, N = 10	Kisii, N = 6
Normal delivery services	16	10	6
BEmONC	16	10	6
CEmONC	2	1	1
Newborn care unit	3	3	0

### Antenatal care services

All the facilities were providing the recommended antenatal care services that includes checking blood pressures, monitoring foetal heartbeat, provision of iron and folic acid supplements, tetanus toxoid immunization, blood test for HIV, and counselling on family planning except Intermittent Preventive Therapy for malaria in pregnancy, which was not provided in any of the six facilities in Kisii County (Table not included).

### BEmONC/CEmONC signal functions

Less than 30% of the facilities provided assisted vaginal deliveries (n = 4/16), two-thirds (11/16) were providing antibiotics for preterm or prolonged PROM (premature rupture of membrane) to prevent infection and half (8/16) were providing corticosteroids in preterm labor. Only two facilities in Kisii had antibiotics for preterm or prolonged PROM (premature rupture of membrane) to prevent infection, one had providing corticosteroids in preterm labor and none could perform assisted vaginal delivers (Table 2).

Administration of oxytocin, hygiene cord care, and thermal protection were provided in all the health facilities. Monitoring and management of labor using partograph and parental administration of oxytocin for treatment of post-partum hemorrhage (IV or IM) was reported in all the facilities except one of the health facilities (15/16). Parental administration of antibiotics (IV or IM) for mothers and removal of retained products of conception services were provided in 14 (88%) and 13 (81%) of the health facilities, respectively. Other services provided were parental administration of magnesium sulphate for management of pre-eclampsia/eclampsia (IV or IM) (n = 12; 75%) and manual removal of placenta (n = 10; 63%). There were only two facilities in the regions providing caesarian section and blood transfusion; one in each of the counties (Table 2).

All the facilities had a place where immediate and exclusive breastfeeding was practiced. Interventions for the management of complications during and after pregnancy and childbirth such as neonatal resuscitation with bag and mask and Kangaroo mother care were carried out in 12 (75%) of the health facilities, whilst 11 (69%) were providing injectable antibiotics for neonatal sepsis (Table 2).

**Table 2:** BEmONC and CEmONC signal functions, N = 16

		County	
Services	Overall, N = 16^1^	Kilifi, N = 10^1^	Kisii, N = 6^1^
Assisted vaginal delivery	4 (25%)	4 (40%)	0 (0%)
Antibiotics for preterm or prolonged PROM (premature rupture of membranes) to prevent infection	11 (69%)	9 (90%)	2 (33%)
Corticosteroids in preterm labor	8 (50%)	7 (70%)	1 (17%)
Administration of oxytocin injection	16(100%)	10(100%)	6(100%)
Monitoring and management of labor using partograph	15 (94%)	10(100%)	5 (83%)
Hygienic cord care	16(100%)	10(100%)	6(100%)
Thermal protection	16(100%)	10(100%)	6(100%)
Parental administration of antibiotics (IV or IM) for mothers	14 (88%)	9 (90%)	5 (83%)
Parental administration of oxytocic for treatment of post-partum hemorrhage (IV or IM)	15 (94%)	10(100%)	5 (83%)
Parental administration of magnesium sulphate for management of pre-eclampsia/eclampsia (IV or IM)	12 (75%)	8 (80%)	4 (67%)
Manual removal of placenta	10 (63%)	7 (70%)	3 (50%)
Removal of retained products of conception	13 (81%)	9 (90%)	4 (67%)
Caesarean section	2 (13%)	1 (10%)	1 (17%)
Blood transfusion	2 (13%)	1 (10%)	1 (17%)
Immediate and exclusive breastfeeding	16(100%)	10(100%)	6(100%)
Neonatal resuscitation with bag and mask	12 (75%)	9 (90%)	3 (50%)
Kangaroo mother care	12 (75%)	8 (80%)	4 (67%)
Injectable antibiotics for neonatal sepsis	11 (69%)	7 (70%)	4 (67%)

^1^n (%)

### Availability of functional equipment

Cord clamp, delivery bed, speculum, infant weighing scale, blood pressure apparatus, clean running water (piped, bucket with tap or pour pitcher), hand-washing soap/liquid soap, and fetoscope were available in all the health facilities. There were few facilities with incubators (n = 4; 25%), vacuum aspirators or D & C kits (n = 5; 31%), suction bulbs, single use (n = 5; 31%), and ultra-sound machines (n = 5; 31%). None of the facilities in Kisii had vacuum aspirators or D & C Kits, suction bulbs, sterilized multi-use and incubators. Other delivery equipment is listed in Table 3.

**Table 3:** Delivery equipment available in Kenya health facilities, N = 16

		County	
Equipment	Overall, N = 16^1^	Kilifi, N = 10^1^	Kisii, N = 6^1^
Examination light (flashlight)	13 (92.9%)	10 (100.0%)	3 (75.0%)
Delivery pack	15 (93.8%)	9 (90.0%)	6(100.0%)
Cord clamp	16(100.0%)	10 (100.0%)	6(100.0%)
Episiotomy scissors	15 (93.8%)	9 (90.0%)	6(100.0%)
Scissors or blade to cut cord	15 (93.8%)	9 (90.0%)	6(100.0%)
Suture material with needle	15 (93.8%)	9 (90.0%)	6(100.0%)
Needle holder functioning	15 (93.8%)	9 (90.0%)	6(100.0%)
Manual vacuum extractor	9 (56.3%)	7 (70.0%)	2 (33.3%)
Vacuum aspirator or D & C kit	5 (31.3%)	5 (50.0%)	0 (0.0%)
Incubator	4 (25.0%)	4 (40.0%)	0 (0.0%)
Blank partograph	15 (93.8%)	10 (100.0%)	5 (83.3%)
Delivery bed	16(100.0%)	10 (100.0%)	6(100.0%)
Resuscitation table (with heat source for newborn resuscitation)	9 (56.3%)	8 (80.0%)	1 (16.7%)
Newborn bag and mask size 1 for term	12 (75.0%)	8 (80.0%)	4 (66.7%)
babies (for newborn resuscitation)			
Newborn bag and mask size 0 for preterm babies (for newborn rescitation)	10 (62.5%)	7 (70.0%)	3 (50.0%)
Electric suction pump (for suction apparatus)	14 (93.8%)	9 (90.0%)	5 (83.3%)
Suction catheter (for suction apparatus) for suctioning newborn	11 (68.8%)	9 (90.0%)	2 (33.3%)
Suction bulb, single use	5 (31.3%)	4 (40.0%)	1 (16.7%)
Suction bulb, sterilizable multi-use	8 (50.0%)	8 (80.0%)	0 (0.0%)
Speculum	16(100.0%)	10 (100.0%)	6(100.0%)
Infant weighting scale	16(100.0%)	10 (100.0%)	6(100.0%)
Blood pressure apparatus (may be	16(100.0%)	10 (100.0%)	6(100.0%)
digital or manual syphygnomanometer with stethoscope)			
Clean running water (piped, bucket with tap, or pour pitcher)	16(100.0%)	10 (100.0%)	6(100.0%)
Hand-washing soap/liquid soap	16(100.0%)	10 (100.0%)	6(100.0%)
Fetoscope	16(100.0%)	10 (100.0%)	6(100.0%)
Doptone or fetal monitorning	11 (68.8%)	10 (100.0%)	1 (16.7%)
Ultrasound machine	5 (31.3%)	3 (30.0%)	2 (33.3%)

### Availability of medicines and commodities

Gentamicin injection and oxytocin were available in all the health facilities whilst only 3/16 of the health facilities had ampicillin powder for injection. These were two facilities in Kilifi and one in Kisii. There was also cold storage for storing oxytocin in all the facilities. IPTp for malaria was available in all the facilities in Kilifi and none in Kisii County. Most of the facilities (n = 14; 88%) were providing injectable artemether/artesunate and oral ACT/AL (e.g. coartem) for malaria treatment. Penicillin for treatment of syphilis was available in 13 (81%) of the health facilities. Oral quinine was available only in one health facility, whereas three facilities had ampicillin powder for injection, and two had injectable quinine. Availability of ampicillin powder for injection, and hydralazine injection was available in most facilities in Kilifi compared to Kisii (Table 4).

**Table 4:** Medicines and commodities available and valid in Kenya health facilities, N = 16

		County	
Medicines	Overall, N = 16^1^	Kilifi, N = 10^1^	Kisii, N = 6^1^
Gentamicin injection	16 (100%)	10 (100%)	6(100.0%)
Ampicillin powder for injection	3 (19%)	2 (20%)	1 (17%)
IPTp for malaria			
Oral SP (Fansdar)	10 (63%)	10 (100%)	0 (0%)
Malaria treatment			
Injectable quinine	2 (13%)	1 (10%)	1 (17%)
Injectable artemether/artesunate	14 (88%)	9 (90%)	5 (83%)
Oral ACT/AL (e.g. coartem)	14 (88%)	8 (80%)	6(100%)
Oral artemeter/artesunate	2 (13%)	0 (0%)	2 (33%)
Oral quinine	1 (6%)	1 (10%)	0 (0%)
Penicillin Rx for treatment of syphilis	13 (81%)	7 (70%)	6(100%)
Hydralazine injection	6 (38%)	5 (50%)	1 (17%)
Oxytocin injection	16 (100%)	10 (100%)	6(100%)
Is the oxytocin stored in cold storage?	16 (100%)	10 (100%)	6(100%)

### General requirements, staffing and guidelines

CEmONC and BEmONC guidelines were available on-site in only three of the health facilities in Kilifi, and none of the facilities in Kisii. Certified health professionals who can perform Caesarean Sections, and who are available 24/7 including weekend/public holidays, were accessible at two facilities, and an anesthetist with similar availability was present at only one facility in Kilifi County and none in Kisii.

There were functional ambulances in five of the health facilities (31%); none in Kisii county. However, all the facilities had access to either ambulances or designated emergency transport services, but only half of them had fuel available on the day of the interview.

Most of the facilities were using either rain water collection (n = 6; 38%) and water piped into the facility (n = 5/16; 31%). Nine of the facilities (56.2%) had water available inside the facilities with the rest either having water outside the facility as ground or water supplied by tanker truck. Of the 16 health facilities (n = 5/16; 31%) had experienced severe water shortage in the previous year before the survey. Of these, three in Kilifi and two in Kisii county. There were three facilities in each of the counties using rainwater collection, one facility in Kisii getting water from an unprotected spring. None of the health facilities in Kisii county were using water from public tap/standpipe nor tube-well/borehole (Table 5).

### Electricity

All the health facilities had solar-powered systems for stand-alone vaccine refrigerators and freezer devices. Fourteen of the health facilities had electricity from the national grid (8 in Kilifi and 6 in Kisii) as the main source of electricity connected to all the departments in these facilities. Of the eight facilities in Kilifi, four had generators as a secondary source of power located in a separate room compared to only one facility in Kisii (Kenyenya sub-county referral hospital). The generators also served the different departments, were functioning, and all five had fuel on the day of the interview. The facilities with the secondary source of power include two of the facilities in Kilifi and one in Kisii that offers CS 24/7 (Table 5).

**Table 5:** Emergency (BEmONC/CEmONC) Services in Kenya health facilities, N = 16

Characteristic		County	
	Overall, N = 16^1^	Kilifi, N = 10^1^	Kisii, N = 6^1^
CEmONC and BEmONC guidelines			
YES, OBSERVED	3 (19%)	3 (30%)	0 (0%)
Health profesional who can perfom CS available 24 h a day including weekends/public holidays			
Yes	2 (13%)	1 (10%)	1 (17%)
Anaesthetist (Dr or nurse) available 24 h a day including weekends/public holidays			
Yes	1 (6%)	1 (10%)	0 (0%)
Functional Ambulance			
Yes	5 (31%)	5 (50%)	0 (0%)
Access to ambulance or emergency transport			
Yes	16(100%)	10(100%)	6(100%)
Fuel available today			
Yes	8 (50%)	4 (40%)	4 (67%)
Main source of water			
Piped into facility	5 (31%)	4 (40%)	1 (17%)
Public tap/standpipe	1 (6%)	1 (10%)	0 (0%)
Tubewell/borehole	1 (6%)	1 (10%)	0 (0%)
Protected Spring	1 (6%)	0 (0%)	1 (17%)
Unprotected Spring	1 (6%)	0 (0%)	1 (17%)
Rainwater Collection	6 (38%)	3 (30%)	3 (50%)
Tanker Truck	1 (6%)	1 (10%)	0 (0%)
Water available from the source within premise			
Inside the facility	9 (56%)	5 (50%)	4 (67%)
Outside facility ground	1 (6%)	0 (0%)	1 (17%)
Not applicable*	6 (38%)	5 (50%)	1 (17%)
Experience of severe water shortage previous year			
Yes	5 (31%)	3 (30%)	2 (33%)
Main source of electricity			
Electricity grid	14(88%)	8 (80%)	6(100%)
Secondary source (generator)	5 (31%)	4 (40%)	1 (2%)
None	2 (13%)	2 (20%)	0 (0)

^1^n (%); *piped into facility/tanker truck

Table 6 shows the number of health care workers that were assigned, employed, or seconded to these facilities during the survey. Despite having a higher number of health facilities, Kilifi had GP in two of the health centers compared to none in Kisii county. There were also MOs in the two hospitals in Kilifi and none in Kisii. Similarly, none of the health facilities in Kisii had a trained midwife to deliver the reproductive maternal newborn and child health services.

**Table 6:** Number of staff assigned, employed, or seconded to the health facilities at the time of the survey

		Cadre of staff assigned/employed/seconded				
	Number of facilities	GP	MO	Nurses	Midwives	CHV
Kilifi						
Dispensary	6	0	0	20	10	210
HC	2	2	0	23	0	139
Hospital	2	10	4	75	0	65
Kisii						
Dispensary	2	0	0	4	0	10
HC	3	0	0	20	0	94
Hospital	1	3	0	30	0	9

*HC: Health center; GP: General practitioner; MO: Specialized medical officer; CHV: Community health volunteer; Hospital: sub-county teaching and referral health facility; Midwives: Trained midwives.*

## Discussion

The study primarily discusses the findings within the context of availability, accessibility, and preparedness to deliver maternal and newborn health care services in two rural sub-counties of Kilifi and Kisii counties. This is to prevent maternal and newborn morbidities and mortalities due to second and third delays [9]. Overall, the study’s findings indicate that although the facilities, particularly health centers and sub-county hospitals, had the necessary equipment, drugs and commodities, there was a limited number of healthcare providers compared to the served population. The workload especially in delivery services, exceed the internationally recommended ratio of 4.5/1000 population of doctors, nurses, and midwives required to achieve sustainable development goals [24]. The inadequate number of health care workers to meet the health care needs of women whenever they arrive at the facilities, is one of the factors contributing to third delay. The findings align with the previous studies that have highlighted the limited supply of health care providers in developing countries [25]. Additionally, Wakaba and colleagues reported a nursing density in public health facilities in Kenya ranging 0.008 to 1.2 per 1000 Kenyan population [26]. Furthermore, a systematic review identified staff shortage as one of the barriers to the third delay in accessing maternal health services [27].

All the 16 facilities surveyed were offering normal delivery and BEmONC services. However, there were only two health facilities in the settings that could offer full CEmONC services as the other hospital was recently upgraded from health center and yet to be equipped, the facilities had only two medical staff members and only one anesthetist available 24/7 to provide maternal services. This implies that there is no given time when two CS can be conducted at the same time since there is only one trained anesthetist available. Any emergency referrals from dispensaries and health centers will have to be delayed for lack of appropriate medical staff. The delay in reaching the health facilities because of limited ambulances and designated transport services with fuel available in a few of them to act during the emergency cases could be a contributor to the number of maternal mortalities experienced in these settings. Unpreparedness of health care professionals and health systems usually during emergency situations, results in maternal deaths, especially in developing countries [25].

The majority of the health facilities performed the BEmONC functions except assisted vaginal deliveries that was performed in only four facilities in Kilifi County and none in Kisii. Manual removal of retained placenta was also performed in fewer health facilities. These findings were similar in a study in Ghana [28]. The availability of BEmONC and CEmONC guidelines were observed only in three health facilities, all of which were in Kilifi County. Guidelines ensure uniformity in the quality of maternal and newborn health service provision. Therefore, a lack of these guidelines in these facilities can be an indicator that might help to understand the quality of care at these health facilities [29]. Kilifi county has had a long history of non-governmental organizations including Kenya Medical Research Institute welcome-trust program as compared with Kisii county which could partly be attributed to the better performance in some of these indicators in Kilifi county.

Antenatal care services were available in all the health facilities except the provision of iPT for malaria which was not available in any of the facilities in Kisii. Kilifi County is classified as a coastal malaria endemic area with a prevalence of 8% while Kisii County is in the highland endemic area with a prevalence of 3% [30]. Lack of facilities not offering iPT in Kisii could be explained by the fact that the facilities are in a low malaria prevalence area since iPT is offered in regions considered to be malaria endemic areas like Kilifi. The World Health Organization (WHO) guidelines for the treatment of malaria recommends a package of interventions for controlling malaria and its effects during pregnancy in areas with moderate to high transmission of Plasmodium *falciparum* that includes use of Insecticide treated nets (ITNs) and administration of IPTp-SP [31]. Kilifi being one of the areas considered to be having moderate transmission rates implemented the guidelines by having oral SP (Fansidar) in all the ten health facilities. Injectable artemether/artesunate, oral ACT/AL, and oral quinine were available in most of the facilities for the treatment of malaria in pregnancy which was in line with the management of malaria in pregnancy [32].

All facilities had refrigerators equipped and powered with solar systems. This guaranteed the availability of immunization services at any point in time even if there was a power outage in the facility. Immunization is a lifesaving and cost-effective medical intervention that reduces childhood morbidity and mortality from disease [33], thus keeping it at the right temperature maintains its potency. The solar systems are maintained by the Kenya Expanded Programme on Immunization (KEPI) which also provide immunization vaccines against the then six killer diseases of childhood, namely tuberculosis, polio, diphtheria, whooping cough, tetanus and measles to all children in the country.

### Limitations

The study covered targeted health facilities within the two rural sub-counties of Kilifi and Kisii among the 47 counties in Kenya. None of the private facilities were included in the sample, thus limiting the generalizability of the findings. The data collected were majorly reported by the health facility in-charge or acting health facility in-charge using quantitative approaches with structured responses and was accurate only as at the time of the survey. This may mask conditions where items were generally unavailable or services lacking before or after the survey. The structured nature of the questionnaire could not assess whether the available equipment were put into correct and appropriate use. The study did not measure some of the indicators in RMCH such as water shortage previous years before the survey, specifications of the cold storage among others. The number of health facilities featured limits the potential for inferential analysis. Finally, due to the nature of our cross-sectional study design, we were unable to evaluate these indicators longitudinally or track their changes over time.

## Conclusion

The facilities were well equipped with the essential drugs and equipment necessary for maternal newborn child health care. However, the infrastructure, including staffing, remains inadequate despite the high number of ANC attendance and deliveries in some of these health facilities. Kilifi County exhibited higher levels of preparedness compared to Kisii County. To fully enhance for the maternal and newborn services, increased investments should be directed towards improving infrastructure and human resources. Additionally, reliable and timely assessment are necessary to track progress, enabling resource allocation based on the need of the health facilities. Conducting key informant interviews with the county health officials would provide a better understanding of critical issues, such as low healthcare worker ratio and inadequate critical infrastructures observed in these facilities.

## Data Availability

The datasets generated and analyzed for this study are available on request from Prof. Marleen Temmerman through the email address: marleen.termmerman@aku.edu. and based on an official data transfer and user agreement sent to the director of the Centre of Excellence in Women and Child Health East Africa at the Aga-Khan University.
